# Root canal morphology of permanent mandibular anterior teeth in a Pakistani population: A cone beam computed tomography assessment

**DOI:** 10.12669/pjms.40.7.8744

**Published:** 2024-08

**Authors:** Saqib Naeem Siddique, Palwasha Babar, Zoha Ghazanfar, Javeria Ahmed Kayani

**Affiliations:** 1 Saqib Naeem Siddique, FCPS. Assistant Professor, Department of Operative Dentistry, University College of Dentistry, The University of Lahore, Lahore, Pakistan; 2 Palwasha Babar, MDS. Assistant Professor, Department of Pediatric Dentistry, FMH College of Medicine and Dentistry, Shadman, Lahore, Pakistan; 3 Zoha Ghazanfar, BDS. House Officer, University College of Dentistry, The University of Lahore, Lahore, Pakistan; 4 Javeria Ahmed Kayani, BDS. House Officer, University College of Dentistry, The University of Lahore, Lahore, Pakistan

**Keywords:** Dental pulp, Pulp canal, Root canal therapy, Tooth abnormalities, Cone beam computed tomography

## Abstract

**Objective:**

To investigate the incidence of accessory canals and the variation in root canal morphology of permanent mandibular incisors and canines in Pakistani population using Cone Beam Computed Tomography (CBCT).

**Methods:**

A cross-sectional study was conducted in University College of Dentistry, The University of Lahore, Pakistan after getting institutional ethical permission from January 2020 to September 2022. The data included records of 111 patients consisting of 444 permanent mandibular incisors and 222 permanent mandibular canines. Accessory root, root canals and Vertucci canal configuration for each tooth was recorded. Data analysis was done using SPSS v20. Descriptive statistics were calculated for each anatomical parameter. Chi-square test was applied to determine association of gender with the presence of accessory roots and root canals.

**Results:**

Among the 111 records evaluated, 48.6% were males and 51.4% were females. No accessory root was found in the central and lateral incisors. However, an accessory root was found in 4.9% of the canines. The incidence of accessory canals in the central incisors, lateral incisors and canines was 18.9%, 25.2% and 10.4% respectively. The most common canal configuration in teeth with accessory canals was Type-III, followed by Type-II and Type-V.

**Conclusion:**

None of the central or lateral incisor showed accessory root while it was detected in 4.9% canines. The frequency of accessory root canal was found to be 18.9%, 25.2% and 10.4% in central incisors, lateral incisors and canines respectively. The most common canal configuration was Type-I, followed by Type-III and Type-II. Type-V, VI and VII were less common.

## INTRODUCTION

A comprehensive understanding of the morphology of the internal root canal system is imperative for endodontic treatment. As the prognosis of the endodontic procedure depends on the complete removal of the pulpal tissue in the root, a thorough knowledge about the presence of any deviation or atypical root canal morphology is very crucial in Endodontics. These anatomical variations present difficulty in negotiating the canals and may result in endodontic failure if the clinician fails to locate, prepare or fill them.^1^ According to a research, as much as 42% of the retreatment in Endodontics has been attributed to missed canal in the teeth.^2^

It is known that the prevalence of the morphological and anatomical variation in the root canals varies with age, gender and ethnicity.^3^,^4^ Mandibular incisors pose a great difficulty in access cavity preparation because of their smaller size. This also makes the location of the accessory canal very difficult.^5^ Globally, there are numerous studies which report the morphology of the root canals in mandibular anterior teeth in different populations. A comprehensive study on 4674 mandibular anterior teeth has reported a prevalence of two root canals in 6.7% of central incisors, 17.4% of lateral incisors and 3% of canines.^6^ A recent meta-analysis reported the prevalence of accessory canals in central incisors, lateral incisors and canines to be 20.4% (15.0%-25.7%), 25.3% (20.0%-30.7%) and 5.9% (4.1%-7.7%) respectively which varies among different ethnic populations.^7^

According to Rahimi et al.,^8^ the incidence of accessory canals in mandibular incisors range from 11.5 to 50% in Iranian population. An Indian study reported a prevalence of 4% accessory canals in mandibular incisors.^9^ In another study conducted in Iraq, the incidence of accessory canals was found to be 52.5% in mandibular central Incisors, 58.4% in mandibular lateral Incisors and 22.7% in mandibular Canines. All incisors were found to be single rooted while 2% of the canines had two roots.^10^

Therefore, it is important to have a detailed knowledge of these anatomical variations in a population to aid in their location and negotiation. There is limited data reporting the anatomical variation in mandibular incisors and canines in Pakistani population. The current study aims to investigate the incidence of accessory canals and the variation in root canal morphology of permanent mandibular incisors and canines in Pakistani population by means of Cone Beam Computed Tomography (CBCT).

## METHODS

This cross-sectional study was conducted in University College of Dentistry, The University of Lahore, Pakistan. The CBCT records included in the study were acquired for diagnostic purposes unrelated to the current study and all patients had provided informed consent for the CBCT scans to be used for research purposes. The current research involved the review and analysis of data from these records. CBCT records of 111 patients from January 2020 to September 2022 were obtained from the Department of Radiology. The CBCT images had been obtained by an experienced radiologist using Planmeca ProMax® 3D Max (Planmeca USA, Inc.); at 90 kV and 5.6mA with an exposure time of 12.09secs with a voxel size of 200um.

### Ethical Approval

The study was approved by Institutional Ethical Review Board, University College of Dentistry (Ref no. UCD/ERCA/21/11dc).

Prior to data collection, the two examiners underwent a training session for calibration regarding reading and recording the observations of CBCT scans. Kappa coefficient score was used to evaluate reliability on 10 CBCT scans that were not included in the study. Inter-examiner reliability was found to be 0.67 and intra-examiner reliability was found to be 0.72 which indicated acceptable agreement.

Untreated, mature permanent mandibular incisors and canines were included in the study. Dentition with congenitally missing anterior teeth, impacted teeth or whereby the root canal anatomy was unclear due to any pathology such as root resorption, pulp canal obliteration or image artifacts were excluded. The data included records of 111 patients consisting of 444 permanent mandibular incisors and 222 permanent mandibular canines. The following anatomical parameters for each tooth were recorded: accessory root, accessory canals, root canal configuration according to Vertucci´s classification.

Data was statistically analyzed using SPSS version 20. Descriptive statistics were calculated for each anatomical parameter. Chi-square test was applied to determine any significant association of gender with the presence of accessory roots and root canals.

## RESULTS

A total of 111 CBCT records that met the inclusion criteria were evaluated among which 48.6% (n=54) were of males and 51.4% (n=57) were of females. Age-wise distribution of the records showed that 18% (n=20) were of <20 years age, 56.8% (n=63) between 21-40 years, 18.9% (n=21) between 41-60 years and 6.3% (n=7) >61 years.

No accessory root was found in the central and lateral incisors. However, an accessory root was found in 4.9% (n=11/222) of the canines [4.5% (n=5) in 33, 5.4% (n=6) in 43]. Three (2.7%) of the patients had two-rooted canines bilaterally while 5 (4.5%) had one two- rooted canine. The accessory canals found in the central incisors were 18.9% (n=42/222) and 25.2% (n=56/222) of lateral incisors. Among the single rooted canines (n=211), an accessory canal was found in 10.4% (22/211) while none of the two-rooted canine (n=11) showed the presence of an accessory canal. The presence of accessory canals with gender was analyzed for possible association. The p-value for central incisors was found to be 0.049, 0.167 with lateral incisors and 0.742 with canines. The gender-wise distribution of the accessory canals in individual set of teeth is summarized in Table-I. A positive association between gender and teeth 41 and 32 was observed with a significant p-value of 0.035 and 0.034 respectively.

**Table-I:** Gender-wise distribution of the accessory canals in individual set of teeth.

Teeth	Accessory Canals			

	Total % (n)	Males % (n)	Females % (n)	p-value
31	19.8 (22)	24.1 (13)	15.8 (9)	0.274
41	18 (20)	25.9 (14)	19.5 (6)	0.035
32	26.1 (29)	35.2 (19)	17.5 (10)	0.034
42	24.3 (27)	29.6 (16)	19.3 (11)	0.205
33	9.9 (11)	7.4 (4)	12.3 (7)	0.39
43	9.9 (11)	11.1 (6)	8.8 (5)	0.638

Type-I configuration was the most common Vertucci classification observed in the mandibular anterior teeth. Type-III configuration was the most frequent canal configuration in teeth with accessory canals followed by Type-II and V. The details of the various canal configurations in individual set of teeth have been summarized in Table-II.

**Table-II:** Vertucci canal configurations observed in mandibular anterior teeth.

Teeth	Vertucci Configuration						

	Type-I % (n)	Type-II % (n)	Type-III % (n)	Type-IV % (n)	Type-V % (n)	Type-VI % (n)	Type-VII % (n)
31	80.2 (89)	0.9 (1)	16.2 (18)	-	1.8 (2)	0.9 (1)	-
41	82.0 (91)	0.9 (1)	15.3 (17)	-	1.8 (2)	-	-
32	73.9 (82)	1.8 (2)	22.5 (25)	-	1.8 (2)	-	-
42	75.7 (84)	1.8 (2)	21.6 (24)	-	0.9 (1)	-	-
33	92.8 (103)	0.9 (1)	5.4 (6)	-	0.9 (1)	-	-
43	93.7 (104)	0.9 (1)	4.5 (5)	-		-	0.9 (1)

The greatest number of bilateral asymmetry was observed in canines. 24.3% (n=27) of central incisors, 23.4% (n=26) of lateral incisors and 34.2% (n=38) showed bilateral asymmetry.

## DISCUSSION

The introduction of CBCT has revolutionized endodontic research as it offers a detailed insight into the internal tooth morphology with minimal radiation exposure to the patient. It is a non-invasive imaging option by which internal anatomic variation of the teeth can be studied in detail.^11^

In the current study, the incidence of accessory canals in central and lateral incisors was found to be 18.9% and 25.2% respectively. This conforms to another study conducted in Pakistan which reported a similar prevalence rate of accessory canals in 23.5% and 29% central and lateral incisors respectively.^12^ The incidence varies greatly in different populations with respective incidence in central and lateral incisors being; 26.2% and 28.8% in Iraqi, 26.3% and 30.8% in Saudian population. On the other hand, a higher prevalence rate of 54% and 71% was reported in Brazilian^13^ and 68% and 63% in Turkish^14^ population.

Mandibular canines are usually single rooted with one canal. However, the incidence of two-roots in the mandibular canines has been reported to be as much as 15%.^15^ The incidence of two roots of canines in our study is 4.9%. This is in conformity with another study conducted in Pakistan which reported an incidence of 5.22%.^16^ In our study, the incidence of accessory canal was found to be 10.4%. This is in agreement with a similar study conducted in India which reported 12.8% incidence of accessory canal in canine.^17^ The most common canal configuration of the accessory canal observed was Type-III followed by Type-II which was very rare (0.9%). A study on Malaysian population reported that 6.1% of mandibular canines had two canals and only Type-II configuration in the accessory canals was observed.^18^ A comprehensive study comparing the Asian and Caucasian ethnic groups reported that it was present in 2.9% and 9.8% of the mandibular canines in the respective populations.^19^

The gender-wise distribution of the accessory canals (Table 1) showed that males had more accessory canals in the incisors compared to females. Similar results were reported by Mashyakhy and Gambarini, who found statistically significant higher number of accessory canals in males.^3^ However, another study conducted on Siberian population reported female predilection for accessory canals in mandibular anterior teeth.^20^ A recent meta-analysis also concluded that the odds of having an accessory canal in incisors is significantly higher in males and is influenced by the demographic factors.^7^

The most common canal configuration was observed to be Type-III, followed by Type-II and Type-V. A Pakistani study reported the incidence of Vertucci’s Type-II in 4%, Type-III in 20.4% and Type-IV in 1.7% of the teeth.^12^ However, they did not include complete Vertucci classification (Type-V, VII and VII) in their evaluation criteria and therefore did not report their incidence. Sinzianna Scarlatescu et al.^21^ evaluated 32 mandibular incisors in Romanian population using clearing technique and reported 65.5% Type-I configuration, 25% Type-III configuration followed by 6.3% Type-II configuration. They also reported Type-VII configuration in 3.1% of cases which was not observed in our population. A recent study^22^ in which micro-CT was used to analyze root canal morphology, reported incidence of Type-II and Type-III configuration to be 6.7% and 3.4% respectively. A Brazilian study reported the incidence of Type-III canal configuration to be as much as 28%.^23^ This shows the anatomical variation present in different populations. A study comparing the Asian and Caucasian populations concluded that Asian have a higher prevalence of Vertucci Type-I configuration while Caucasians displayed a higher incidence of multiple root canal system morphologies.^19^

### Limitations

The findings of the current study need to be considered in light of several methodological limitations. It is a single-center study with a limited sample size. The convenience sample taken in this study does not entirely represent the Pakistani population. However, the study paves way for additional research on the subject.

### Note

Chat GPT was used to improve Grammar and scientific writing.

## CONCLUSION

A comprehensive knowledge about the root canal morphology and the aberrant anatomies is essential for the success of endodontic treatment. None of the incisors showed accessory root while it was detected in 4.9% canines. The frequency of accessory root canal was found to be 18.9%, 25.2% and 10.4% in central incisors, lateral incisors and canines respectively. The most common canal configuration was Type-I, followed by Type-III and Type-II. Clinicians should be aware of these anatomical variations in order to ensure successful endodontic treatment of these teeth.

### Authors’ contributions:

**SNS:** Conceived and designed the study, supervised the data collection, performed data analysis, reviewed the final manuscript.

**PB:** Helped in analysis and interpretation of data, drafted the manuscript and coordinated contributions from co-authors, finalized the manuscript.

**ZG and JAK:** Data collection, reviewed manuscript for finalization.

All authors agree to be accountable for the accuracy and integrity of this work.
